# Mechanically, the Shoot Apical Meristem of Arabidopsis Behaves like a Shell Inflated by a Pressure of About 1 MPa

**DOI:** 10.3389/fpls.2015.01038

**Published:** 2015-11-26

**Authors:** Léna Beauzamy, Marion Louveaux, Olivier Hamant, Arezki Boudaoud

**Affiliations:** ^1^Laboratoire Reproduction et Développement des Plantes, INRA, Centre National de la Recherche Scientifique, ENS de Lyon, UCB Lyon 1, Université de LyonLyon, France; ^2^Laboratoire Joliot-Curie, Centre National de la Recherche Scientifique, ENS de Lyon, Université de LyonLyon, France; ^3^Institut Universitaire de FranceParis, France

**Keywords:** shoot apical meristem, epidermis, turgor pressure, atomic force microscopy, indentation, mechanical modeling

## Abstract

In plants, the shoot apical meristem contains the stem cells and is responsible for the generation of all aerial organs. Mechanistically, organogenesis is associated with an auxin-dependent local softening of the epidermis. This has been proposed to be sufficient to trigger outgrowth, because the epidermis is thought to be under tension and stiffer than internal tissues in all the aerial part of the plant. However, this has not been directly demonstrated in the shoot apical meristem. Here we tested this hypothesis in Arabidopsis using indentation methods and modeling. We considered two possible scenarios: either the epidermis does not have unique properties and the meristem behaves as a homogeneous linearly-elastic tissue, or the epidermis is under tension and the meristem exhibits the response of a shell under pressure. Large indentation depths measurements with a large tip (~size of the meristem) were consistent with a shell-like behavior. This also allowed us to deduce a value of turgor pressure, estimated at 0.82±0.16 MPa. Indentation with atomic force microscopy provided local measurements of pressure in the epidermis, further confirming the range of values obtained from large deformations. Altogether, our data demonstrate that the Arabidopsis shoot apical meristem behaves like a shell under a MPa range pressure and support a key role for the epidermis in shaping the shoot apex.

## 1. Introduction

Plants generate leaves and/or flowers during their entire life thanks to the activity of a group of dividing cells at the tip of each branches, the shoot apical meristem (SAM). This tissue contains a stem cell niche at its center, from which cells are continuously recruited in growing organs. This process is highly regulated, as shown for instance by the highly ordered pattern of organ initiation along the stem, also called phyllotaxis. The molecular basis for organogenesis at the SAM has been investigated in much detail, and a rather complex gene network is now available in which signaling molecules (peptides, miRNA, hormones) control the spatio-temporal pattern of key transcription factors (Ha et al., 2010; Sablowski, 2010). This regulation translates into shape changes, notably by the modification of the cell wall mechanical properties (Milani et al., 2013; Ali et al., 2014). In particular, the local softening of cell walls by the addition of expansin can trigger organogenesis in the SAM (Fleming et al., 1997). Auxin-dependent pectin demethylesterification has also been shown to induce wall softening and promote organogenesis in the SAM (Peaucelle et al., 2011; Braybrook and Peaucelle, 2013). Recently, organogenesis has been associated with a local auxin-dependent randomization of microtubule arrays in the SAM epidermis, arguably leading to isotropic cellulose microfibril orientation in the cell wall and outgrowth (Sassi et al., 2014). Importantly, these studies often assume that the epidermis of the SAM is limiting for growth, implying that the epidermis is under tension. How true is such an assumption?

The concept of tissue tension dates back to famous nineteenth century botanists such as Hofmeister and Sachs (Peters and Tomos, 1996). Typical experiments involved making cuts through plant organs. If the gap left after the cut remains open, this reveals that the tissue was under tension before the cut. If two tissues are pulled apart and their dimensions change, the tissue that shrunk was in tension before being separated. Such experiments showed that the epidermis is in tension in maize coleoptiles (Kutschera et al., 1987) or in sunflower hypocotyls (Peters and Tomos, 2000). Cracking of the epidermis also occurs spontaneously in Arabidopsis mutants: In hypocotyls (Bouton et al., 2002) and in stems (Maeda et al., 2014). How epidermal tension is generated is still debated (Peters and Tomos, 1996), though a possible explanation is that turgor pressure is mostly supported by the epidermis because it is stiffer than internal tissue layers (Boudaoud, 2010). Indeed, direct mechanical measurements using extensometry showed a high epidermal stiffness in sunflower hypocotyls (Hejnowicz and Sievers, 1996), in mature tulip stems (Kutschera et al., 1987), and in mature leaves in a broad range of species (Onoda et al., 2015). The theory of epidermal control of growth was proposed based on such data (Kutschera, 1992; Kutschera and Niklas, 2007): The growth of the inner tissues is restrained—and therefore controlled—by the epidermis. This theory received mechanistic support by the finding that brassinoid perception solely in the epidermis was sufficient to obtain wild-type looking Arabidopsis plants (Savaldi-Goldstein and Chory, 2008).

However, despite the range of available data, this theory has been questioned, notably concerning the supposedly passive role of the inner layers (Peters and Tomos, 1996). For example in chimeric leaves where the epidermis is genetically distinct from inner tissues, the final size cannot be explained by considering exclusively the epidermal layer (Marcotrigiano, 2010). In the framework of this theory, the axial elongation of stems would be ascribed to the mechanical anisotropy of epidermal walls associated with transverse cellulose microfibrils. However, cellulose microfibrils are found to be axial in surface walls (Baskin and Jensen, 2013). In Arabidopsis hypocotyls, it is the inner face of epidermal cells that shows clear alignment of microtubules perpendicular to the growth direction, not the outer face (Chan et al., 2011; Crowell et al., 2011), predicting transverse cellulose at the inner face of the epidermis. This reinforces the view of both the inner and outer layers playing an active role in determining the final shape of the plant.

In the shoot apical meristem, a relatively similar debate is taking place: cuts in sunflower capitulum (Dumais and Steele, 2000) or in tomato (Reinhardt et al., 2003) are consistent with the idea that the epidermis of the meristem center would be under tension, though the periphery of the meristem in asteraceae can be concave leading to the prediction of orthoradial compressive stress (Dumais and Steele, 2000). Osmotic treatments in the tomato SAM revealed a good correlation between the mechanical properties of the epidermis and organogenesis (Kierzkowski et al., 2012). Outer cell walls are significantly thicker than inner cell walls in tomato (Kierzkowski et al., 2012). However, wall thickness might not be well-correlated with wall stiffness, as wall composition/remodeling may vary independently. Auxin-dependent pectin demethylesterification was found to be initiated in inner tissues during organogenesis (Peaucelle et al., 2008). Consistent with this finding, it was shown that the subepidermal layer (L2) softens before the epidermis (L1) during organogenesis using indentation with small and large probes (Peaucelle et al., 2011). Softening the epidermis by overexpressing *PECTIN METHYLESTERASE 5* in the epidermis did not alter organogenesis, while overexpressing the same gene in the entire meristem promoted organogenesis, thus questioning the role of the epidermis in triggering outgrowth in the SAM (Peaucelle et al., 2011). Here we propose to use indentation and modeling to test whether the meristem behaves or not like a thin shell under pressure.

## 2. Materials and methods

### 2.1. Plants

We used *p35S::LTi6B-GFP* and *p35S::GFP-MBD* (Marc et al., 1998) Arabidopsis lines (Ws-4 ecotype) for the nano-indentation experiments. For the atomic force microscopy experiments we used one meristem from the *p35S::LTi6B-GFP* Arabidopsis line and another one from the *pCLV3::GFPer* Arabidopsis line (Landsberg *erecta* ecotype, as in Milani et al., 2014). Seeds were sown on soil, kept at 4°C during 48 h, then grown in short day conditions (8 h light at 19°C ; 16 h night at 17°C) during 4 weeks and transferred 2–3 weeks in long days conditions (16 h day at 21°C; 8 h night at 19°C) until they bolted.

### 2.2. Shoot apices

**Figure 1:**
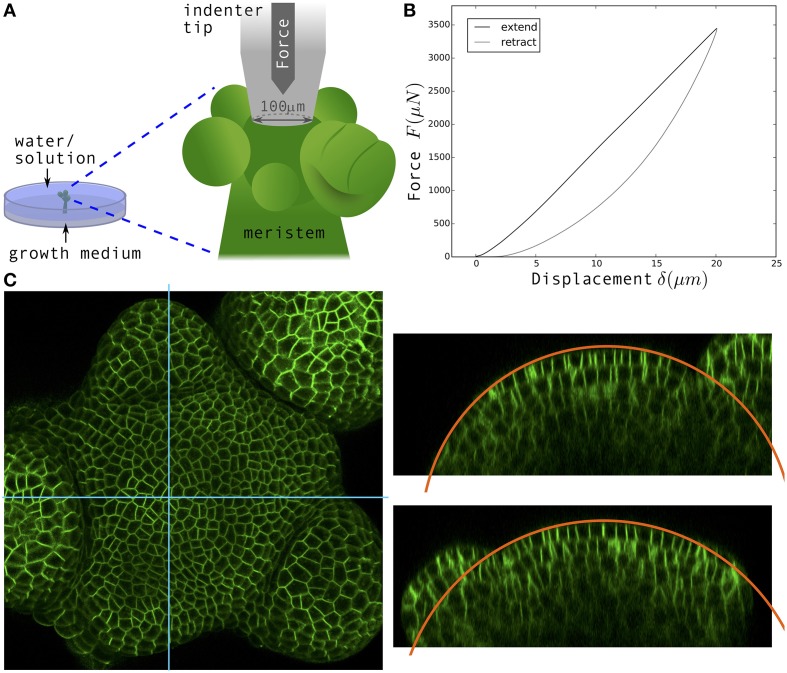
**Nano-indentation measurements and confocal imaging of dissected shoot apical meristems. (A)** Schematic of the experimental setup. Dissected meristems are inserted in solid medium and immersed in water or solution for indentation measurements. **(B)** Typical Force-displacement curve obtained. Black: Approach, Gray: Retract. **(C)** Left: Surface projection of confocal image stack of a *p35S::LTi6B-GFP* shoot apical meristem viewed from the top; the fluorescent signal indicates cell membranes. Right: Orthogonal views corresponding to the blue sections. Orange circular lines: Fits of the surface in order to determine the radii of curvature.

Shoot apices were prepared on the day before mechanical measurements to enable water potential equilibration (Diaz-Pérez et al., 1995) before experiments. The top 2 cm of the inflorescence stem were cut and as many organs as possible were dissected out to allow the indenter tip to access the meristem surface. Apices were then stuck in a small Petri dish filled with medium (see Figure 1A). In order to keep apices growing (Fernandez et al., 2010), we used the Arabidopsis apex culture medium (ACM: 2.2 g/l Duchefa Biochemie-MS basal salt mixture without vitamins, 1% sucrose; pH adjusted to 5.8 with KOH, and 1.6% agarose added); the medium was supplemented with vitamins (1000X stock solution : 5 g Myo-inositol Sigma, 0.05 g Nicotinic acid Sigma, 0.05 g Pyridoxine hydrochloride Sigma, 0.5 g Thiamine hydrochloride Sigma and 0.1 g Glycine Sigma in 50 mL water) and 200 nmol benzyladenine (BAP). If necessary, meristems were mechanically stabilized by extra drops of ACM without vitamins and BAP. Dissected meristems were kept in a phytotron in long days conditions (Panasonic Versatile Environmental Test Chamber, 16 h day at 21°C; 8 h night at 19°C, synchronized with growth culture chambers) during the night before the measurements.

### 2.3. Solutions

All measurements in turgid conditions were done in ultrapure water, both for nano-indentation and atomic force microscopy (AFM). The hypertonic solution, used for the nano-indentation measurements in flaccid conditions, was prepared by dilution of 3.64 g of mannitol in 50 mL DPBS 1X. The osmolarity of the resulting solution was measured with an osmometer (Osmomat 030, Gonotec) and found to correspond to an osmotic pressure of 1.8 MPa. Meristems were immersed in the solution for 20 min prior to measurements and imaging. In order to reduce the effects of solutes in the medium, the shoot apex was rinsed with excess water/solution just before the start of experiments.

### 2.4. Confocal microscopy and determination of surface mean curvature

Plants were imaged in water prior to their first indentation, and immediately after their second indentation in the plasmolysis solution. 1024 × 1024 pixels images with slices every 1 µm were acquired on a upright confocal microscope (LSM 700, Zeiss), with a water-dipping 40x lens. The fluorescent signal indicated either the plasma membrane (*LTi6B-GFP*) or microtubules (*GFP-MBD*). An example of confocal image is visible in Figure 1C. Surface projections were obtained with MerryProj software (de Reuille et al., 2005). In the shoot apex, curvature is heterogeneous at cell scale (see Kwiatkowska, 2006, for instance), but our analysis only required the large-scale curvature of each meristem, at the same scale as indentation depths. We used orthogonal views to determine meristem radii (the inverse of curvatures) by manually fitting the surface with a circle (in the Fiji software): see the orange circular arc in Figure 1C for an example. The mean curvature of the meristem κ_*M*_ was defined as the average between the curvatures in 2 perpendicular directions. The radius of curvature *r* = 1∕κ_*M*_ was in the range 50–100 µm.

### 2.5. Nano-indentation

All 62 measurements (37 shoot apices) were done in water or in hypertonic solution. Nano-indentation experiments were performed with a TI 950 TriboIndenter and its associated extended stage that enables vertical displacements to be higher than 5 µm (Hysitron). We used a truncated cone tip with a disk-shaped flat end of ~100 µm diameter (we took the exact value 96.96 µm for all calculations). Unlike in atomic force microscopy (Milani et al., 2013), this tip almost covered the entire surface of the meristem (see Figure 1A). In the software we chose a displacement-controlled function, allowing us to impose a maximum displacement and a specific rate. A first 10 µm displacement ramp was followed by a pause of 5 s just above the sample surface and then a second ramp with 20 µm maximum displacement at a speed of 4 µm s^−1^ (5 s of approach, 5 s of retraction). The first ramp helped finding the sample surface and only the second ramp was analyzed here; the pause was intended for the sample to recover from any viscous-like deformation due to the first ramp. In most instances, the two ramps yielded the same quantitative curves.

Raw force-displacement curves (Figure 1B) were analyzed with a homemade Python script. The approach curve has two regimes: non-linear at small depth and linear at larger depth. We therefore performed two types of fits: a Hertz fit in the region 0–7 µm, and a linear fit in the region 18–20 µm (see Section 3 and Supplementary Material for the justification of these intervals); the functional form of the fits is shown in **Figure 3A**.

### 2.6. Atomic force microscopy

All AFM experiments and analyses were performed using the same methodology as in Beauzamy et al. (2015) with the following minor modifications. The meristems were prepared as described above. The value of the cell wall thickness was set to 190 nm, according to TEM images of meristem slices (Cloarec and Traas, unpublished data), but note that the values of pressure deduced are rather insensitive to the exact wall thickness (Beauzamy et al., 2015; Weber et al., 2015). Indentation depths reached 1.2 µm, which is greater than the height of the top wall of a single cell (typically 800 nm), so that our measurements were sensitive to neighboring cells and we therefore needed to compute a multicellular curvature (see Beauzamy et al., 2015, for a justification). To compute this curvature in practice, we considered the area of the meristem defined by all cells probed and approximately one additional row of cells (in practice we took 110% of the area of the cells probed). We fitted this area of the AFM height map with a 2nd order polynomial and computed the corresponding curvatures (see **Figure 5A** for an example of 3D view of the surface of the meristem). We also used the QNM (Quantitative Nanomechanical Mapping) imaging mode to distinguish the cell contours (Milani et al., 2014), see for example the LogDMTmodulus map on **Figure 5B** (the green line indicates the studied cells on this meristem). Each studied cell was probed at 3 different locations, with 2–3 repetitions per location. We analyzed in total 510 force-depth curves from 65 cells in two meristems.

## 3. Results

### 3.1. The meristem viewed as a homogeneous tissue

**Figure 2:**
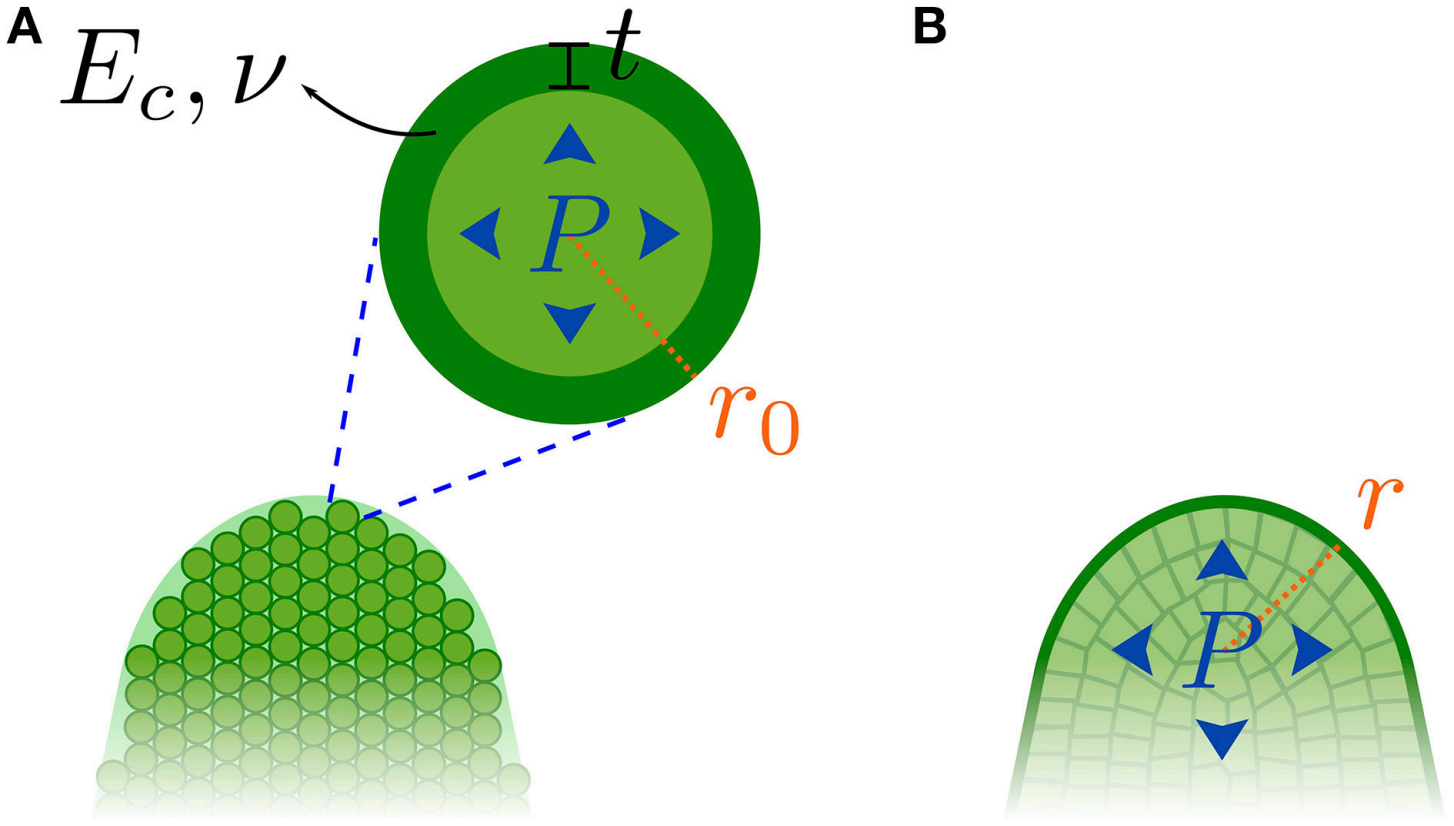
**Two mechanical models for the shoot apical meristem. (A)** A homogeneous cellular aggregate. **(B)** A shell under pressure (negligible internal walls).

We first investigated whether the shoot apical meristem (SAM) could be considered as a homogeneous tissue. To do so, we performed indention experiments and interpreted the resulting force-depth curves so as to obtain elastic moduli. Such tissue-scale Young's moduli can be related to cellular parameters using the mechanical model developed in Nilsson et al. (1958) for a homogeneous cellular aggregate with internal pressure as a representation of a homogeneous plant tissue (see Figure 2A).

This model deduces the Young's modulus *E* of the tissue from the Young's modulus of individual cell walls *E*_*c*_, the thickness of the cell walls *t*, the Poisson's ratio ν of the cell walls, the radius *r*_0_ of the cells, and the internal pressure *P*, assumed to remain constant.

This representation results in a tissue with a linear-elastic mechanical behavior, characterized by a large-scale elastic modulus *E* (equation as reformulated in Niklas, 1992):

(1) $$\begin{array}{rcl} E_{turgid} & = & 3P\left(1+\frac{7-5\nu }{20\left(1+\nu \right)}\right)+E_{plasmo};\\ \;E_{plasmo} & = & \frac{3E_{c}t\left(7-5\nu \right)}{10r_{0}\left(1-\nu^{2}\right)} \end{array}$$

This equation shows two contributions to the modulus of a turgid tissue, *E*_*turgid*_: The first is proportional to turgor pressure, *P*; the second is uniquely due to the cell walls and is equal to the modulus, *E*_*plasmo*_, of the tissue when it is flaccid (i.e., when the pressure vanishes). Accordingly, measuring the large-scale moduli when the tissue is turgid and when it is flaccid should enable the deduction of pressure knowing Poisson's ratio (we used the default value ν = 0.5 for incompressible materials, a value of ν = 0.3 would only shift the results by 5%).

**Figure 3:**
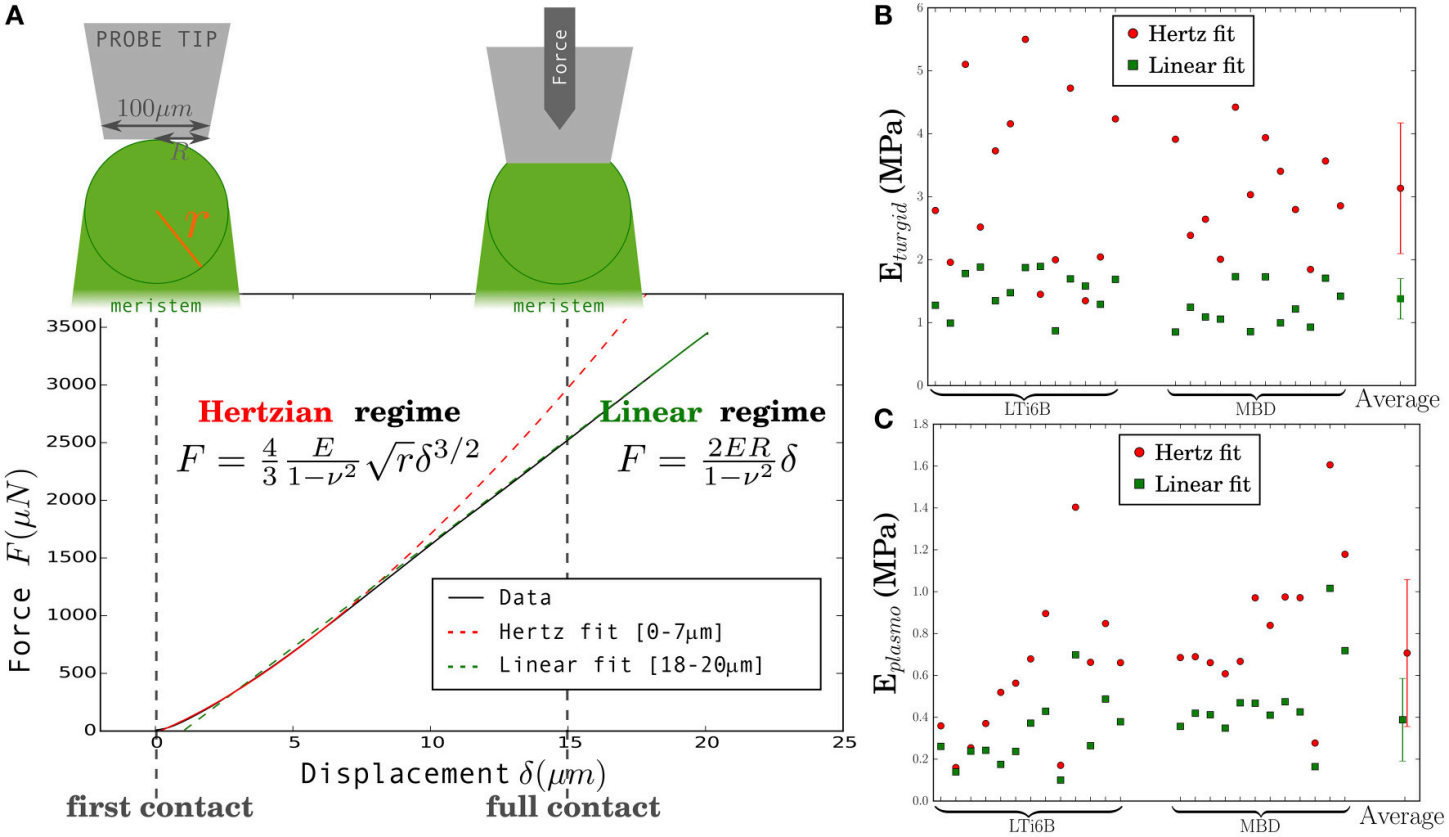
**Extraction of a global meristem Young's modulus *E* from the two regimes of the Force-displacement curve. (A)** Typical Force-displacement curve separated into: Hertzian regime (incomplete contact between the probe and the sample) and linear regime (full contact between tip and sample), and corresponding relationships between force, *F*, and displacement, δ. **(B,C)** Calculated Young's modulus *E*_*turgid*_ **(B)** and *E*_*plasmo*_ **(C)** for 25 meristems, in turgid and flaccid states, respectively: Hertzian (red circles) and linear (green squares) regimes. The vertical bars span the average plus or minus one standard deviation. Shoot apices are shown in the same order in **(B,C)**. LTi6B and MBD indicate the marker line the meristem belongs to.

We extracted elastic moduli from indentation experiments. In order to choose appropriate models from contact mechanics (Johnson, 1987), we considered the geometry of the tissue. Meristems are dome-shaped, so the indented part can be approximated by a half-sphere of radius *r* (deduced from the mean curvature, *r* = 1∕κ_*m*_), while we used a flat and wide tip (100 µm diameter). Thus, at small indentation depth, the situation is similar to the well-known Hertzian indentation of a flat sample with a sphere, see Figure 3A. At high indentation depths, the tip reaches full contact with the sample and the contact surface does not increase anymore with indentation depth. The relationship between force, *F*, and indentation depth, δ, becomes linear (Johnson, 1987), see Linear regime on Figure 3A. The transition between these two regimes depends on the radius of curvature of the meristem, *r*. Based on the values of *r*, we estimated that the 0–7 µm region of the force-depth curve should exhibit a Hertzian behavior, whereas the 18–20 µm region should correspond to the linear regime (see Supplementary Material). If the meristem were homogeneous, then these two regimes would yield the same value of Young's modulus.

We performed indentation experiments on 2 different fluorescent plant marker lines (*p35S::LTi6B-GFP* and *p35S::GFP-MBD*) so as to enable the measurement of SAM radius of curvature with confocal microscopy. The two lines behaved similarly in all our measurements.

For each of the 25 turgid meristems studied, the Young's modulus *E* was extracted by fitting each regime of the force-depth curve to the appropriate model. Figure 3B shows all results. The values range from 1.34 to 5.50 MPa and from 0.85 to 1.89 MPa for the Hertzian and the linear regimes, respectively. The corresponding mean values are 3.13±1.13 and 1.37±0.35 MPa (Here and elsewhere we give the mean value and the standard deviation).

Following the first indentation, each meristem was plasmolyzed, and we extracted the flaccid Young's modulus from a second indentation experiment. The results are shown in Figure 3C. This elastic modulus respectively range from 0.16 to 1.60 MPa and from 0.10 to 1.01 MPa for the Hertzian and the linear regimes. The respective mean values are 0.71±0.35 MPa and 0.39±0.20 MPa.

In both turgid and plasmolyzed conditions, the modulus extracted from the first part of the curve (Hertz fit) was higher than that extracted from the last part (linear fit; Figures 3B,C). The clear difference between the values of elastic modulus, *E*, obtained from the 2 fits indicates that we must reconsider the hypothesis of the meristem being a homogeneous linearly-elastic material. The value obtained from the Hertz fit could be impacted by errors due to the optical estimation of the curvature or by changes in curvature from the turgid to the flaccid state, but an error of 10% on the radius of curvature, *r*, (or a change of 10% in *r*) would only lead to an error of 5% on the modulus, *E*. A possible explanation is that the meristem is not linearly elastic. As internal pressure would be expected to increase upon compression while the modulus is smaller for larger depths, such non-linearity cannot be ascribed to turgor, but only to the cell wall and so should be enhanced in flaccid meristems. In contrast, the ratio between moduli (*E*_*turgid*_ ∕ *E*_*plasmo*_) is observed to be smaller in flaccid meristems than in turgid ones. Therefore, the higher modulus obtained from small depth indentation suggests that the meristem has a stiffer outer layer, instead of being homogeneous. This stiffer layer could be the external cell wall as it was observed to be thicker than internal walls in tomato (Kierzkowski et al., 2012), but could also be the whole epidermal layer (L1).

**Figure 4:**
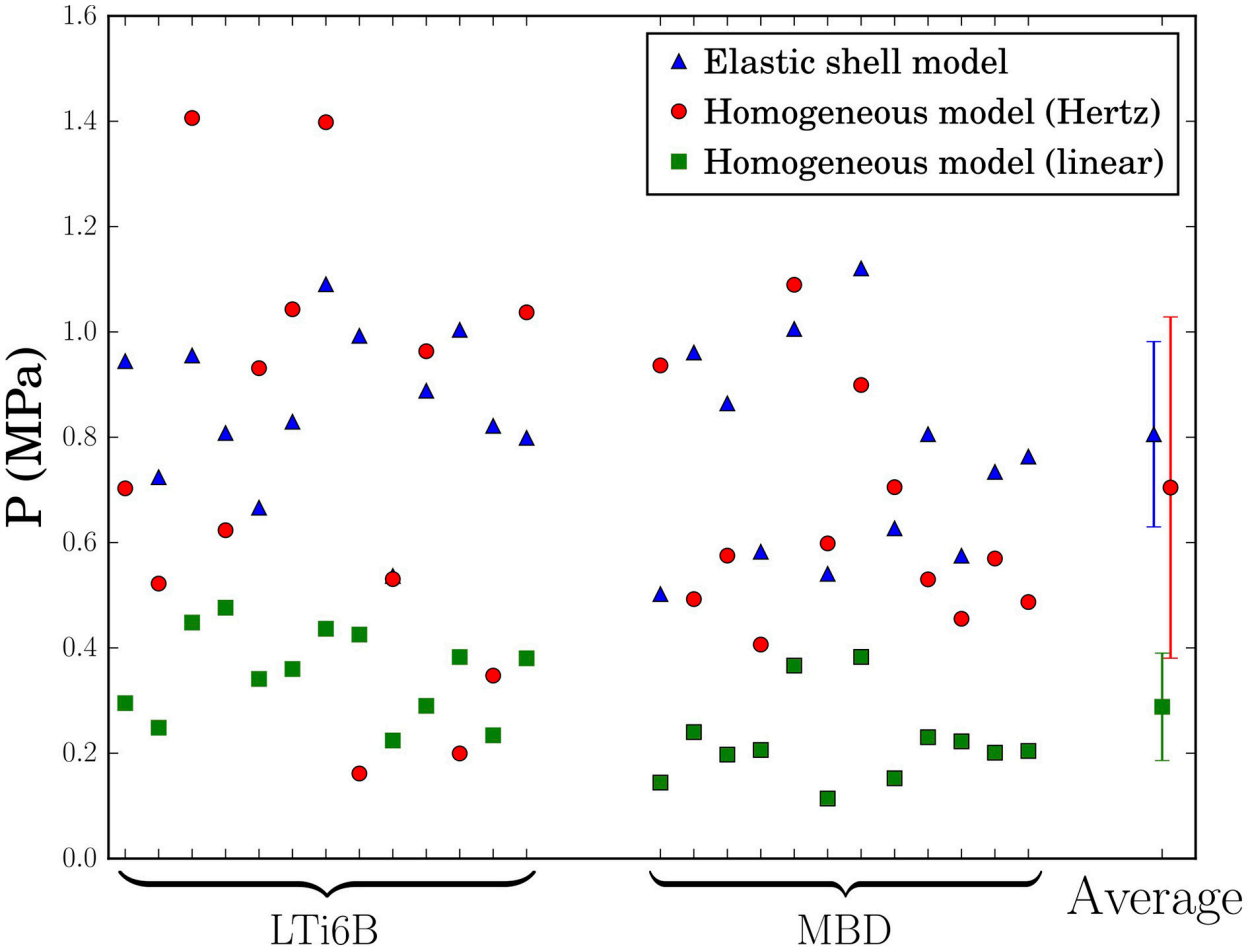
**Values of pressure in the shoot apices deduced from indentation experiments using 3 different models:** pressurized shell (blue triangles), small depth indentation of a homogeneous solid (red circles), or large depth indentation of a homogeneous solid (green squares). LTi6B and MBD stand for the Arabidopsis marker lines. The vertical bars span the average plus or minus one standard deviation.

In order to further support this conclusion, we extracted the pressure from each indentation regime using the values of modulus in both turgid and flaccid states and Equation (1), under the assumption that the corresponding model is applicable. These results are shown in Figure 4 and will be discussed below.

### 3.2. The meristem viewed as a shell under pressure

As suggested by the analysis above, we now consider the hypothesis that the meristem is a shell under pressure (Figure 2B). We use the results of previous theoretical work showing that, when indenting pressurized elastic shells, the force *F* applied is proportional to the displacement δ (Vella et al., 2012). At large indentation depths, the slope, *k*, of the force-displacement curve is directly proportional to the internal pressure, *P*:

(2) $$\begin{array}{r} k=\frac{\pi P}{\kappa_{M}}=\pi rP, \end{array}$$

κ_*M*_ = 1∕*r* being the mean curvature of the shell and *r* the corresponding radius of curvature. The main conditions for the validity of the model are that (i) indentation depth is sufficiently larger than the thickness of the shell, (ii) that the pressure is sufficiently high, and (iii) that turgor pressure is constant. Therefore, this model cannot describe force curves at small depths or for flaccid meristems. Here, we use for the linear fit indentation depths greater than 18 µm, which is clearly larger than the shell thickness be it the outer cell walls or the L1 layer. Finally, the assumption that the pressure is constant is not a limitation because it has been shown (Weber et al., 2015) that the pressure variations are small when, instead, the volume is assumed to be constant.

The values of pressure deduced are presented in Figure 4, together with the values deduced from the first approach. Using the pressurized shell model, the small indentation depth homogeneous model and the large indentation depth homogeneous model, we found that the pressure values ranged respectively from 0.50 to 1.12 MPa, from 0.16 to 1.40 MPa, and from 0.11 to 0.47 MPa, with average values at 0.80±0.18 MPa, 0.70±0.32 MPa, and 0.29±0.10 MPa. We used the coefficient of variation (standard deviation divided by average) to assess the models. Indeed, equal validity of the models would lead to the same coefficient of variation through the propagation of measurement errors. Here the coefficient of variability takes the values 0.22, 0.46, and 0.36, respectively. The lower value of variation coefficient for the shell model shows that the slope is correlated with curvature, as expected from Equation (2), and indicates that it better describes the SAM mechanics than the two other models.

Interestingly, the shell model gave pressure values that are in the same range as in the homogeneous model when using the small-depth fit to deduce elastic moduli. As the small depth fit extended from 0 to 7 µm which is slightly larger than L1 thickness, we expect it to be mostly sensitive to the mechanics of the L1 and therefore to probe a rather homogeneous system. Therefore, the approach considering a homogeneous meristem has likely more validity at small depth (consistently with the intermediate value of variation coefficient) and we expect the corresponding values of pressure to be comparable to the values obtained with the shell model.

Finally, in order to refine the value of turgor pressure deduced from the shell model, we performed additional indentation experiments on 12 turgid SAMs, increasing the total number of studied apices to 37. All these experiments led to a pressure of 0.82±0.16 MPa, in agreement with the value previously obtained (see Supplementary Figure 2).

### 3.3. Pressure measurements at cellular scale

**Figure 5:**
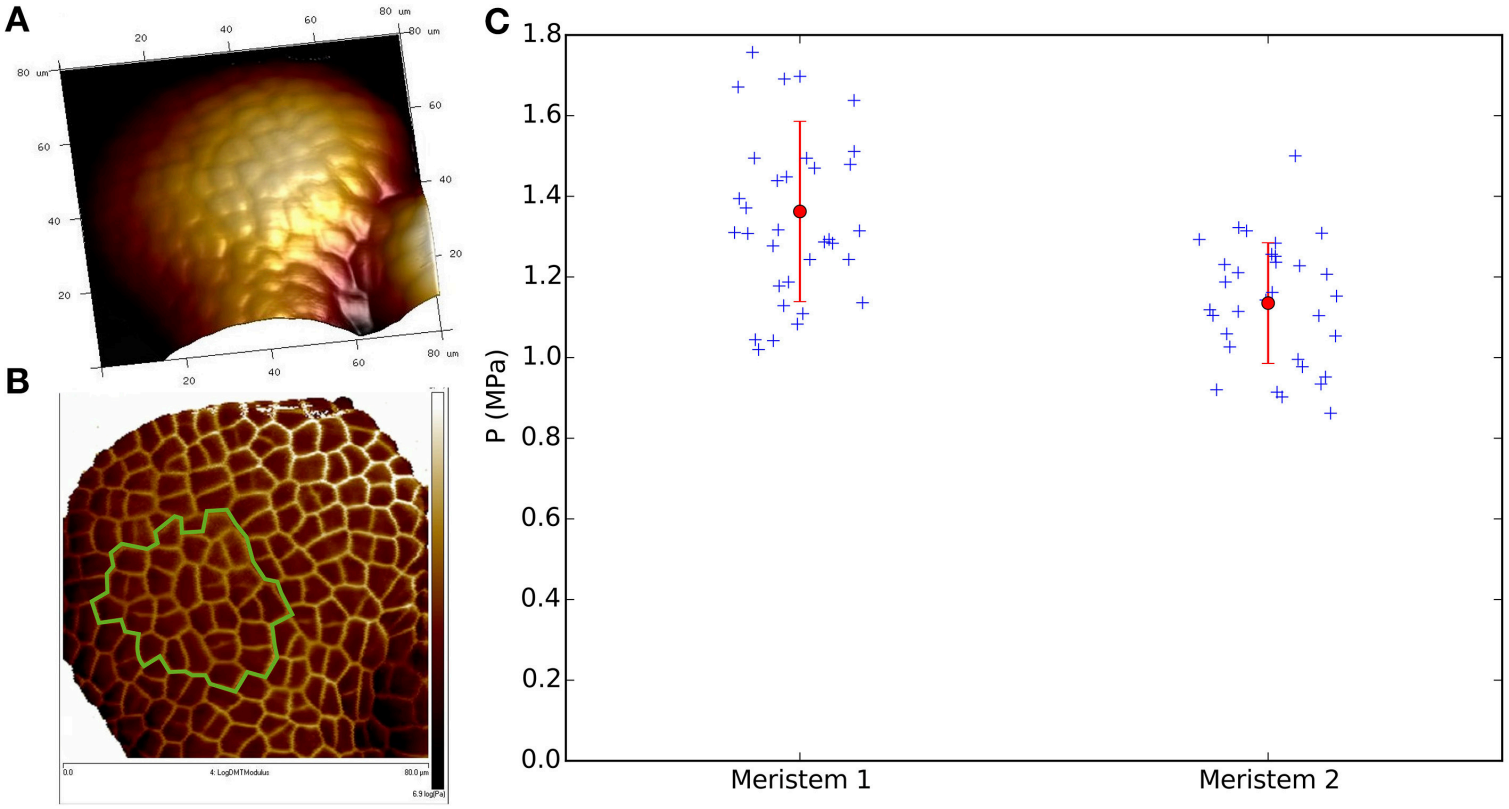
**The pressure in meristematic cells from atomic force microscopy. (A,B)** 80 × 80 μm AFM scan of a meristem (Meristem 2). Emerging organs are visible at the bottom. **(A)** 3D view of meristem surface (Height channel). Lighter regions are higher than darker ones. **(B)** Image of the same meristem highlighting cell contours (LogDMTModulus channel). The green line indicates the location of the 32 neighboring cells probed in this meristem. **(C)** Pressure values of 34 (Meristem 1 = *p35S::LTI6b-GFP* in Ws-4) and 32 (Meristem 2 = *pCLV3::GFP* in Ler) cells, located close to or inside the central zone. Each blue cross corresponds to one specific cell (average over the 3 repetitions × 3 locations per cell). The mean value per meristem and its corresponding SD are plotted in red.

To further confirm the order of magnitude of pressure obtained from the shell model, we next used atomic force microscopy to deduce the pressure in the epidermis. We chose this approach because cells in the shoot apex are too small for the classical pressure probe to be used. We followed the methodology established in Beauzamy et al. (2015). Briefly, we find the local pressure in the epidermis by scanning the surface of the meristem to obtain height maps and by performing local indentations on cells. We probed 65 cells (from 2 meristems) located close to or within the central zone, yielding 510 curves to analyze. The region probed in one meristem is shown in Figure 5B. The pressure measurements results are shown in Figure 5C.

The mean pressure value for all the studied cells was respectively 1.36±0.22 MPa and 1.14±0.15 MPa for meristem 1 and 2. The slight differences between these two meristems could come from the low sample size and/or the different ecotypes (Ws-4 vs. Ler); indeed we found lower values in another ecotype, Col-0, in the approximate range 0.5–1 MPa (Supplementary Figure 3). Nonetheless, in all cases the mean pressure value of each meristem is of the same order of magnitude as the values obtained in the previous section with the elastic shell model (see Figure 4). The mean pressure measured in the epidermis is therefore comparable to the global pressure value of the meristem seen as a shell under pressure.

## 4. Discussion

In this paper we aimed at discriminating between two mechanical models of the shoot apical meristem, using indentation experiments whereby Arabidopsis shoot apical meristems (SAMs) are flattened by a disk-ended flat tip. We first considered a homogeneous cellular solid model and deduced the tissular elastic moduli from small-depth indentation and from large-depth indentation. The modulus from small depth was significantly larger, suggesting that the meristem is inhomogeneous and that the outer layers are stiffer. We therefore considered a pressurized shell model and found that it better described the SAM mechanics. This does not imply that internal layers are completely negligible, but rather that the outer part of the meristem is significantly stiffer than internal tissues.

Our results highlight the key role of the epidermis in the SAM, either all the L1 epidermal cell layer or only its outer cell walls, which acts as a shell that sustains the inner pressure. An important consequence is that the epidermis of the SAM is in tension. This is consistent with observations that the pressure puts the epidermis under tension, even leading to strain-stiffening (Hejnowicz and Sievers, 1996; Niklas and Paolillo, 1997; Kierzkowski et al., 2012), reinforcing the view of the epidermis as a protective skin. Following theses studies, we cannot exclude that more complex non-linear models are more appropriate to fully describe the shoot apex. We note however that the pressurized shell model is insensitive to whether cell walls are non-linearly elastic.

Together with previous studies, our work suggests that remodeling of cell walls in the epidermis is necessary for organogenesis. In the future, it would be interesting to use our global indentation approach to characterize the mechanics of flower primordia in wild type and in plant lines in which cell wall properties are altered or modified. Indeed, outgrowth correlates with expansin activity in the outer tissues (Fleming et al., 1997; Pien et al., 2001) or with auxin-dependent pectin demethylesterification in the L1 (Peaucelle et al., 2011; Braybrook and Peaucelle, 2013). Accordingly, auxin patterning in the L1 is necessary and sufficient for organogenesis (Kierzkowski et al., 2013). However, organogenesis also requires pectin demethylesterification in the L2 subepidermal layer (Peaucelle et al., 2011), which implies that the L2 is not negligible mechanically, at least during organogenesis. Indeed, intuitively, internal layers cannot be completely negligible. Alternatively, the shell model may apply to the whole tunica (L1 + L2) of the SAM (though the thin shell approximation would not fully apply to the tunica).

The number of layers in the tunica of SAMs varies between and within species. For instance, the number of tunica layers in a pelagornium species is positively correlated with the meristem radius of curvature (Wegner, 2000). Assuming little variations in turgor pressure, the tension in the outer stiffer shell is proportional to the radius of curvature. Consequently, it is tempting to propose that the number of layers in the tunica depends on this tension. Mechanistically, periclinal tension would favor anticlinal divisions, which are characteristic of each of the layers in the tunica. Consistent with this, the radius of curvature of floral meristems in Arabidopsis (Milani et al., 2014) is 2–3 times smaller than in SAMs, which correlates with the reduction of the number of tunica layers from 2 (L1 + L2) to 1.

Accordingly, surface geometry varies as the tissue is displaced from the dome-like (positive Gaussian curvature) meristem center to the periphery of the meristem, which becomes either dome-like (with smaller radii of curvature) when incorporated in organs primordia or cylinder-like (zero Gaussian curvature) along the stem. These drastic changes in geometry are also accompanied by changes in cell mechanics (Milani et al., 2013). Future work should incorporate this dynamics in order to build a comprehensive mechanical model of the shoot apex.

It is well known that the stiffness of herbaceous plants strongly depends on turgor pressure (Beauzamy et al., 2014). Consistent with this, we found that apparent elastic moduli of SAMs are decreased by a factor of 2–3 upon plasmolysis. The pressurized shell model yielded values of pressure of about 0.8 MPa, and we confirmed this order of magnitude using a recently developed method based on atomic force microscopy (Beauzamy et al., 2015). Our work provides two methods to estimate turgor pressure, either locally in the epidermis or globally in the whole SAM. Both methods involve indentation and interpretation using a mechanical model. The differences between the values of pressure in the epidermis and the global value of pressure might be ascribed either to the incomplete validity of one of the two models or to a higher turgor in the L1. We find values of pressure around 1 MPa, that are within the range of values measured in other tissues (Beauzamy et al., 2014). We might overestimate turgor pressure because it is expected to increase upon tissue compression, but the resulting error was predicted to be small (Weber et al., 2015), or because apices in water have an increased turgor. Making the assumption that there is no osmoregulation in the tomato SAM, the concentrations of isotonic and plasmolyzing solutions yield an estimate of 0.5 MPa for turgor pressure (Kierzkowski et al., 2012), which is the same range as the values obtained here. Our approach allowed us to estimate pressure by indenting only turgid SAMs, which will be helpful for future experiments. In addition, as the pressure probe is technically not suited for the small cells of the shoot apex, our approach is essential to estimate turgor pressure in this tissue.

A higher tension in the epidermis might also have implications on sensing. Cortical microtubules were shown to orient according to the direction of main tension in the epidermis (Hamant et al., 2008; Jacques et al., 2013; Sampathkumar et al., 2014), and the pressurized shell model of the SAM was hypothesized to support this conclusion (Hamant et al., 2008). A stiffer epidermis that is limiting for growth would make it a key tissue to respond to internal and external signals or perturbations (Ingram, 2008; Savaldi-Goldstein and Chory, 2008).

While we provide here evidence that the pressurized shell model is good approximation of the mechanics of the shoot apex, it is unlikely that this generalizes to all plant organs. On the one hand, it has been shown that the leaf epidermis is stiffer than internal layers in leaves (Onoda et al., 2015). On the other hand, predicted axial mechanical stress in the epidermis of sunflower hypocotyls is incompatible with a pressurized cylinder model Hejnowicz and Sievers (1996). More strikingly, lateral roots emerge through the outer layers of the primary root, suggesting that these layers are relatively soft (Vermeer and Geldner, 2015).

Finally, we note that morphogenesis depends on mechanical parameters of the tissues, such as turgor pressure or cell wall extensibility. A number of computational studies have started addressing how the cellular control of these parameters is translated into organ shape (Ali et al., 2014; Boudon et al., 2015). However, many of these parameters need to be determined through biophysical experiments (Milani et al., 2013). Our work provides a way to assess the hypotheses behind models of the shoot apical meristem or of floral meristems, as well as values of turgor pressure. More generally, the combination of molecular, biophysical, and computational studies will likely be instrumental to advance our understanding of morphogenesis.

## Author contributions

LB and AB designed the research; LB and ML performed the research; LB, ML, OH, and AB analyzed the data; LB, OH, and AB wrote the paper.

## Funding

This work was supported by the European Research Council (Starting Grant PhyMorph 307387 to AB) and by a PhD grant from ARC3 Environnement, Région Rhône-Alpes.

### Conflict of interest statement

The authors declare that the research was conducted in the absence of any commercial or financial relationships that could be construed as a potential conflict of interest.
