# Supplementary material for: Mechanically, the Shoot Apical Meristem of Arabidopsis Behaves like a Shell Inflated by a Pressure of About 1 MPa
Source: Front Plant Sci. 2015 Nov 26;6:1038. doi: 10.3389/fpls.2015.01038 (PMC4659900; doi:10.3389/fpls.2015.01038)
Supplement: Supplementary file 1 [file Presentation1.PDF]

Supplementary Material: Mechanically, the shoot apical meristem of Arabidopsis behaves like a shell inflated by a pressure of about 1 MPa

Léna Beauzamy, Marion Louveaux, Olivier Hamant and Arezki Boudaoud *
*Correspondence:
Arezki Boudaoud
arezki.boudaoud@ens-lyon.fr

1 ESTIMATION OF THE DEPTH OF FULL CONTACT BETWEEN THE PROBE AND THE SAMPLE

Based on simple geometry (see Fig.1), we estimated value of indentation depth $\delta_{fullcontact}$ at the tip reaches in full contact with the shoot apical meristem as

$$\delta_{fullcontact} = r - \sqrt{r^2 - R^2}, \tag{1}$$

where r is the radius of the meristem, and R the radius of the probe ($R = 48.48$ μm). Note that this estimate does not account for the deformation of the meristem on the sides, which makes full contact occur at smaller depths. Therefore this estimate should be taken as an upper bound; for instance, in the case of a Hertzian contact with a homogeneous sample, $\delta_{fullcontact}$ is overestimated by a factor of 2 (K.L. Johnson, *Contact Mechanics*, Cambridge University Press, 1987).

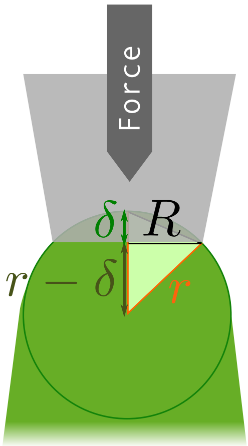

Supplementary Figure 1. Schematic of the indentation when the tip reaches full contact with the shoot apical meristem.

In order to ease comparisons between samples, we sought a unique range where Hertz/Linear fits can be reasonably applied. Since the full contact point is underestimated, we chose 0 to 7 μm depth for the Hertzian fit, as 7 μm is smaller that the minimal $\delta_{fullcontact}$ in Table 1, and 18 to 20 μm depth for the

Supplementary Table 1. Mean curvature radii and corresponding full contact indentation depth $\delta_{fullcontact}$ of all the meristems studied, at turgid and plasmolyzed states. All rows are deduced from confocal imaging, except for the 2 last ones which result from AFM scans.

meristem	r_{turgid}	$\delta_{fullcontact}$	r_{plasmo}	$\delta_{fullcontact}$
LTi6B-01	55.9	28.0	66.2	21.1
LTi6B-02	57.3	26.7	65.6	21.4
LTi6B-03	74.4	18.0	90.7	14.1
LTi6B-04	95.9	13.2	111.6	11.1
LTi6B-05	83.3	15.6	104.1	12.0
LTi6B-06	73.2	18.4	80.0	16.4
LTi6B-07	70.7	19.2	89.5	14.3
LTi6B-08	78.5	16.8	94.6	13.4
LTi6B-09	66.7	20.9	69.1	19.9
LTi6B-10	78.5	16.8	100.5	12.5
LTi6B-11	64.8	21.8	73.6	18.2
LTi6B-12	64.6	21.9	64.2	22.1
LTi6B-13	86.9	14.8	94.4	13.4
MBD-01	46.2	-	48.4	-
MBD-02	53.7	30.6	62.6	23.0
MBD-03	53.9	30.4	54.2	30.0
MBD-04	64.5	22.0	54.2	30.0
MBD-05	70.8	19.2	84.8	15.2
MBD-06	65.1	21.6	74.3	18.0
MBD-07	63.4	22.5	68.5	20.1
MBD-08	65.4	21.5	65.2	21.6
MBD-09	62.1	23.3	67.0	20.7
MBD-10	66.4	21.0	81.2	16.1
MBD-11	95.6	13.2	117.7	10.7
MBD-12	76.5	17.3	78.7	16.7
MBD-13	77.2	17.1	-	-
MBD-14	62.7	23.0	-	-
MBD-15	67.8	20.4	-	-
MBD-16	63.7	22.4	-	-
MBD-17	81.8	15.9	-	-
MBD-18	76.3	17.4	-	-
MBD-19	83.1	15.6	-	-
MBD-20	80.5	16.2	-	-
MBD-21	84.2	15.4	-	-
MBD-22	81.9	15.9	-	-
MBD-23	84.3	15.3	-	-
MBD-24	69.9	19.5	-	-
AFM-01	88.2	14.5	-	-
AFM-02	90.1	14.2	-	-

linear fit. The values of the determination coefficient R^2 for the linear fits, always greater than 0.99, was consistent with the linear regime being reached in this region.

We further checked the robustness of our results using two approaches. (i) Reducing the depth intervals (though this makes the fits less reliable) did not affect the values of moduli or of slopes. (ii) Fitting the whole 0 to 20μm depth interval with a functional form that generalizes the Hertzian and the linear fit was satisfactory in about 2/3 of the cases and yielded the same quantitative results; in 1/3 of the cases, the whole-depth fit was unsatisfactory (low determination coefficient), often because of an inflection point in the curve. We therefore only used the 0-7μm and 18-20μm intervals to obtain the data shown in the main figures.

2 THE MERISTEM VIEWED AS A SHELL UNDER PRESSURE

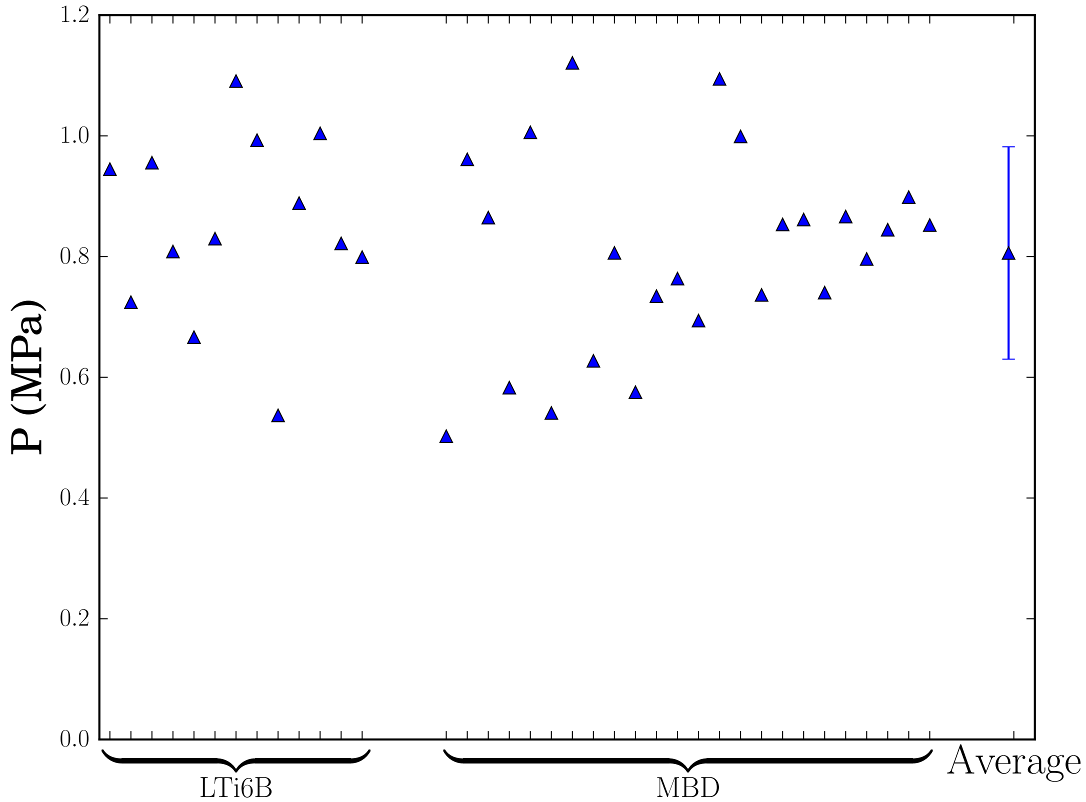

Supplementary Figure 2. Values of pressure in SAMs deduced from indentation experiments using the pressurized shell model. LTi6B and MBD stand for the Arabidopsis marker lines. 12 additional meristems are located at the right of the graph, whereas the 25 others are in the same order as in Figure 4. The vertical bars span the average plus or minus one standard deviation. Mean value: 0.82 ± 0.16 MPa.

3 LOCAL VALUES OF PRESSURE FROM ATOMIC FORCE MICROSCOPY

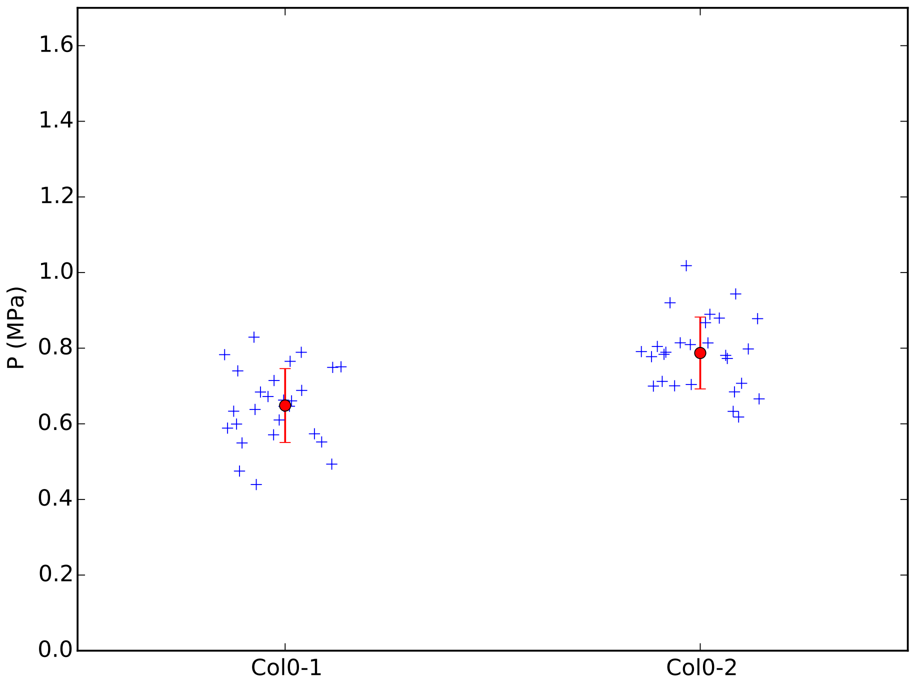

Figure 3. The pressure in meristematic cells from atomic force microscopy (additional wild type meristems in the Col-0 background). Pressure values of 27 cells in each meristem, located close to or inside the central zone. Each blue cross corresponds to one specific cell (average over the 3 repetitions at one location per cell). The mean value per meristem and its corresponding SD are 0.65 ± 0.10 MPa and 0.79 ± 0.09 MPa, respectively (both plotted in red).
